# Supplementary material for: Abundant and metabolically flexible bacterial lineages underlie a vast potential for rubisco-mediated carbon fixation in the dark ocean
Source: Genome Biol. 2025 Jun 16;26:167. doi: 10.1186/s13059-025-03625-3 (PMC12168267; doi:10.1186/s13059-025-03625-3)
Supplement: Supplementary file 2 — Additional file 2: Supplementary figures. Supporting figures for the analyses presented in this study. [file 13059_2025_3625_MOESM2_ESM.docx]

### **Supplementary Figures**


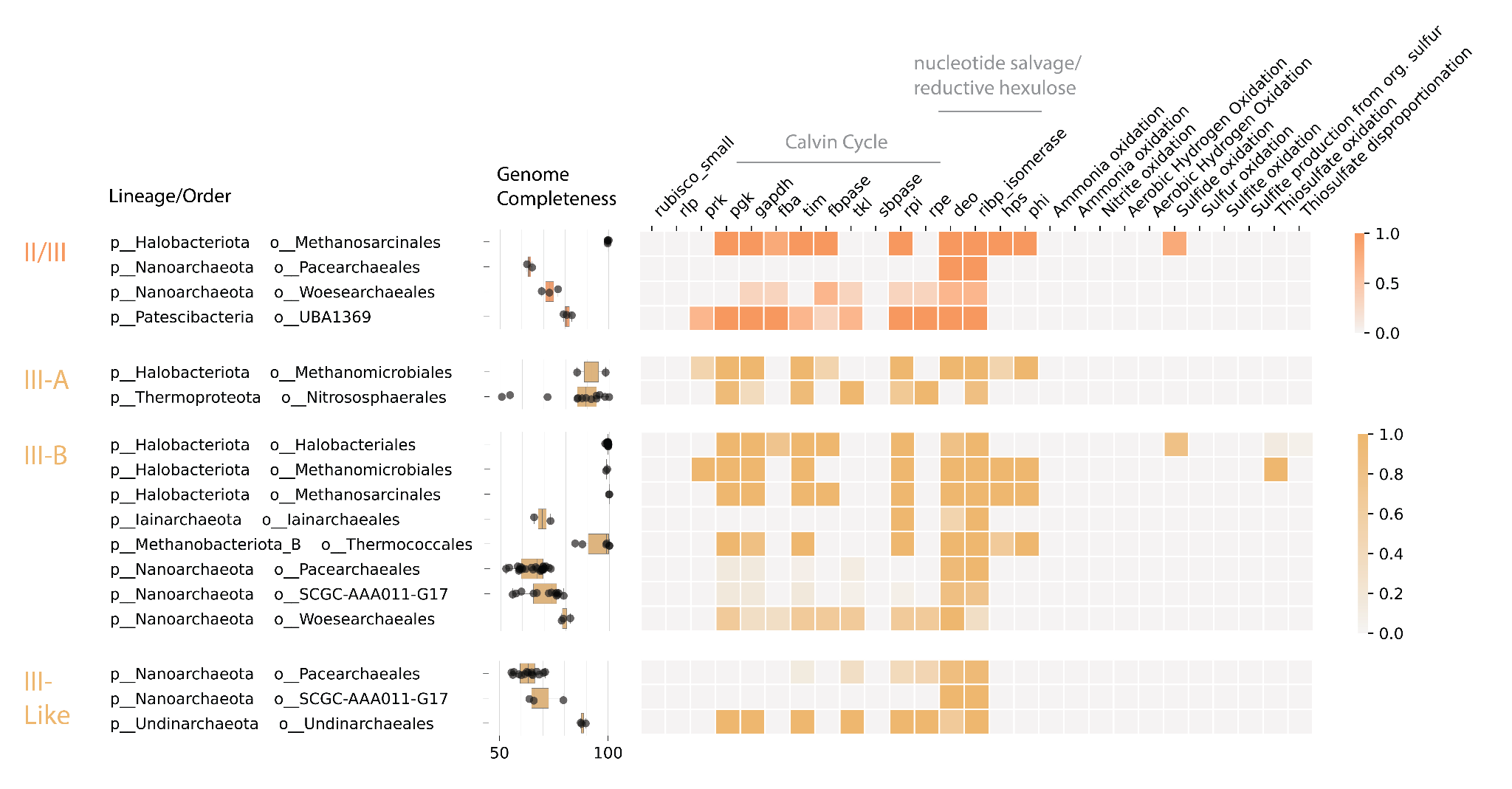


**Fig. S1.** Characteristics of rubisco-encoding organisms (REOs) with the form II/III or form III-related enzyme types. Organisms were clustered into species groups (95% ANI) and were aggregated at the order level. **a)** For each order-level lineage, the completeness of representative genomes for each species group, and fraction of representative genomes that encode various rubisco-associated genes as well as those involved in oxidation of sulfur or nitrogen compounds for energy gain. Only those order-level lineages with more than one species group are displayed.


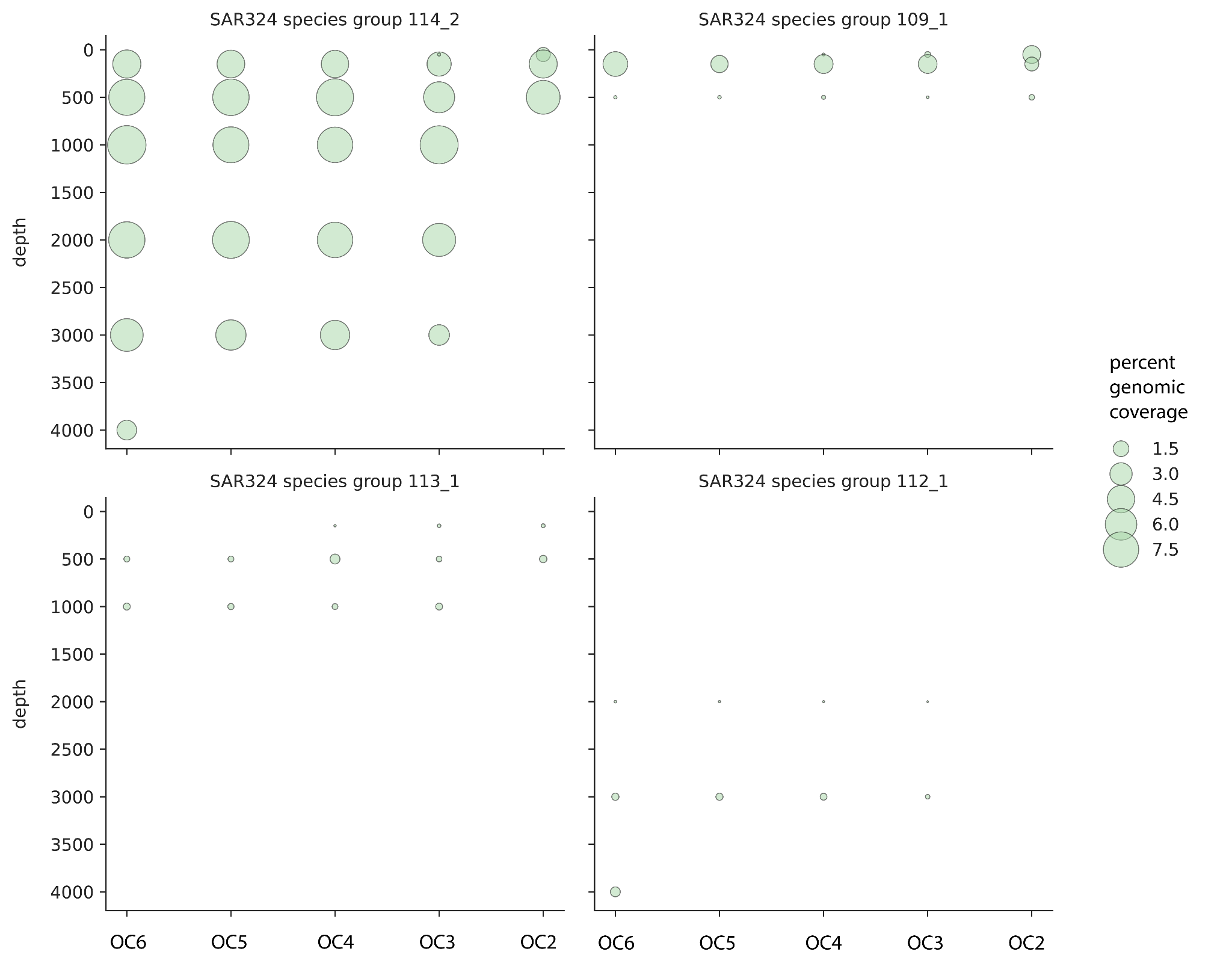


**Fig. S2.** Distribution and abundance of SAR324 species groups with the form I rubisco across the OC1703A transect (coastal California). Dotplots represent 5 sites (OC2-6) over a span of 300 km from shore sampled across depths. Size of dots represent the relative abundance (calculated as the percentage of total genomic sequencing coverage).


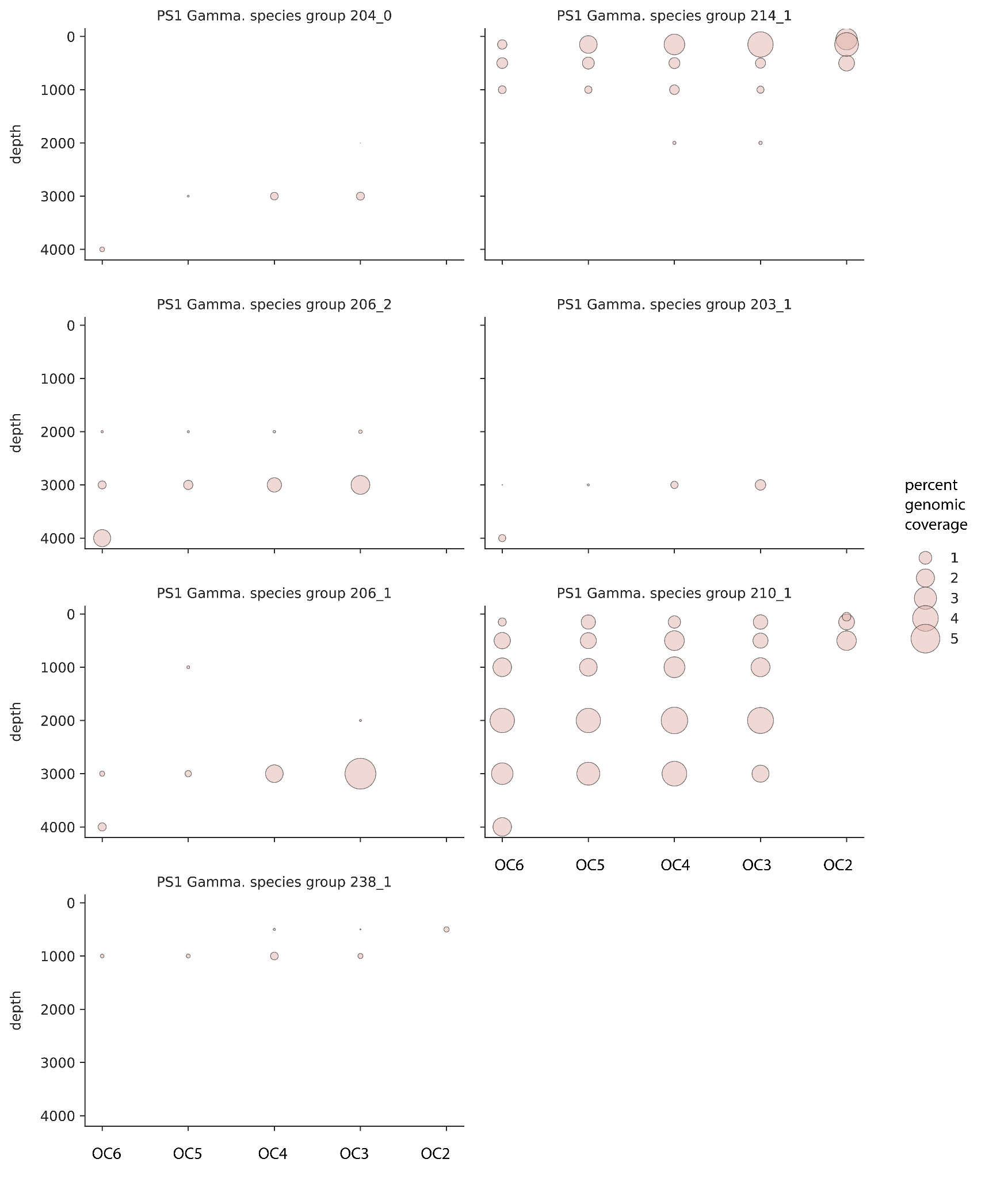


**Fig. S3.** Distribution and abundance of PS1 (Gammaproteobacteria) species groups with the form II rubisco across the OC1703A transect (coastal California). Dotplots represent 5 sites (OC2-6) over a span of 300 km from shore sampled across depths. Size of dots represent the relative abundance (calculated as the percentage of total genomic sequencing coverage).


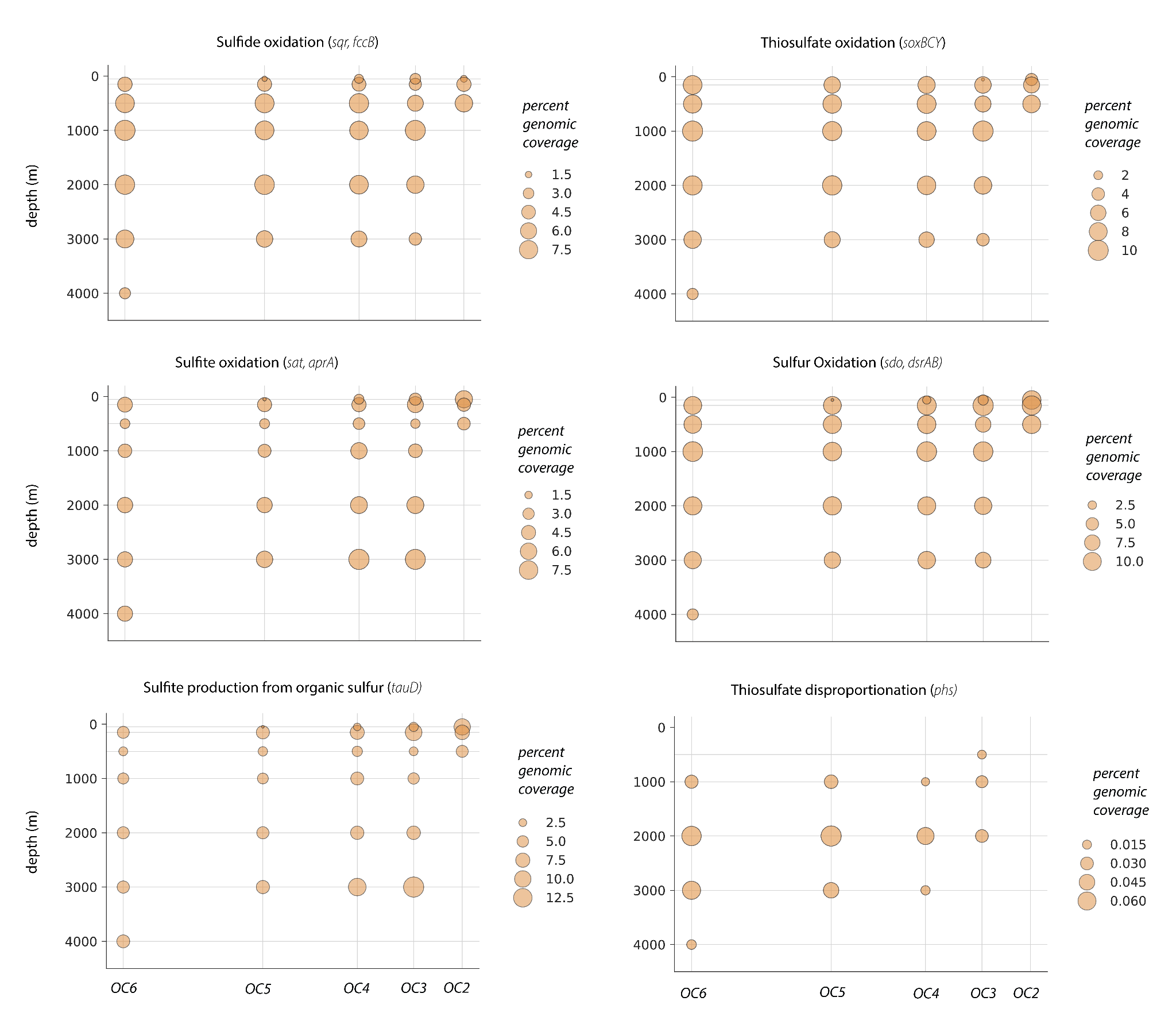


**Fig. S4.** Distribution and abundance of REOs with the genetic capacity for various sulfur oxidation pathways across the OC1703A transect (coastal California). Dotplots represent 5 sites (OC2-6) over a span of 300 km from shore sampled across depths. Size of dots represent the summed relative abundance (calculated as the percentage of total genomic sequencing coverage) of all organisms encoding a given gene/pathway.


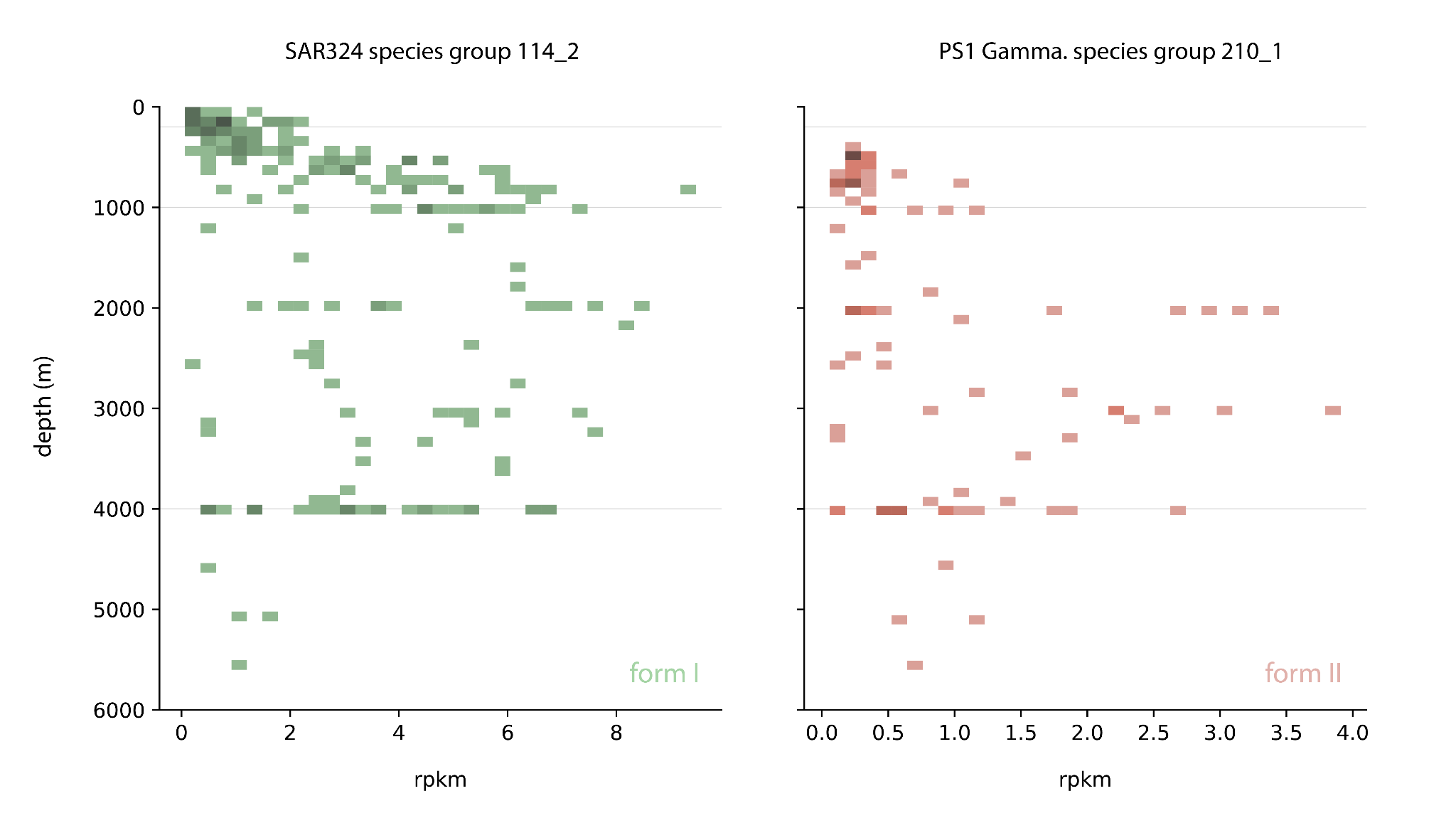


**Fig. S5.** Global distribution and abundance of two rubisco-encoding species groups from the SAR324 and PS1 Gammaproteobacteria orders, respectively. Relative abundance is expressed as RPKM (reads per kilobase million). Shaded cells indicate the detection of one or more organisms in a given depth/RPKM bin, with hue intensity indicating the density of observations in that bin.


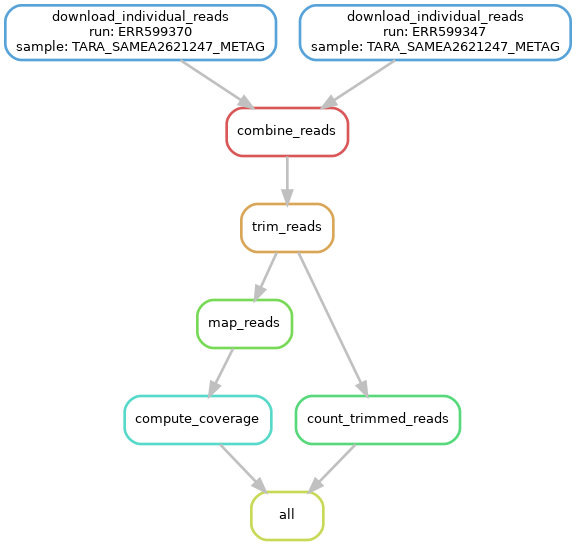


**Fig. S5.** Conceptual overview of the computational workflow (implemented in Snakemake) used to align reads from global water column metagenomes to the REO genome set.


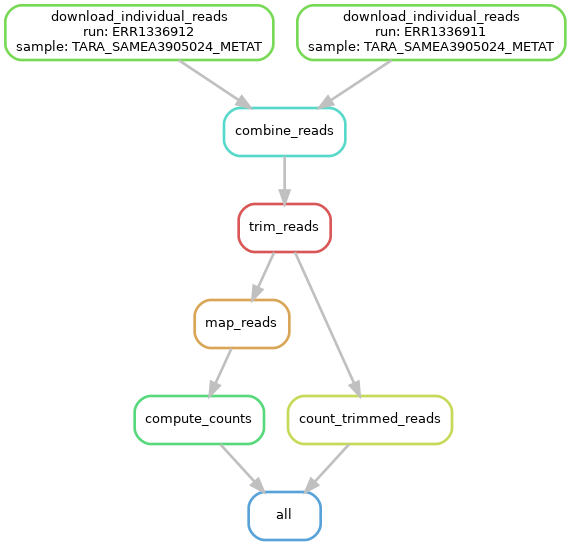


**Fig. S6.** Conceptual overview of the computational workflow (implemented in Snakemake) used to align reads from global water column metatranscriptomes to the REO genome set.
